# Activation of Olfactory Receptors on Mouse Pulmonary Macrophages Promotes Monocyte Chemotactic Protein-1 Production

**DOI:** 10.1371/journal.pone.0080148

**Published:** 2013-11-21

**Authors:** Jing Jing Li, Hock L. Tay, Maximilian Plank, Ama-Tawiah Essilfie, Philip M. Hansbro, Paul S. Foster, Ming Yang

**Affiliations:** Centre for Asthma and Respiratory Disease, School of Biomedical Sciences and Pharmacy, Faculty of Health and Medicine, The University of Newcastle and Hunter Medical Research Institute, Callaghan, New South Wales, Australia; Boston University, United States of America

## Abstract

**Background:**

Emerging evidence suggests that non-olfactory tissues and cells can express olfactory receptors (ORs), however, the exact function of ectopic OR expression remains unknown. We have previously shown in mouse models that a unique cooperation between interferon-γ (IFN-γ) and lipopolysaccharide (LPS) drives the activation of pulmonary macrophages and leads to the induction of pathogenic responses in the respiratory tract. Further, through gene array studies, we have shown that activation of macrophages by these molecules results in the selective expression of a number of ORs. In this study, we validated the expression of these ORs in mouse airway and pulmonary macrophages in response to IFN-γ and LPS (γ/LPS) stimulation, and further explored the effect of odorant stimulation on macrophage function.

**Methodology/Principal Findings:**

OR expression in airway and pulmonary macrophages in response to IFN-γ, LPS or γ/LPS treatments was assessed by microarray and validated by q-PCR. OR expression (e.g. OR622) on macrophages was confirmed by visualization in immunofluoresence assays. Functional responses to odorants were assessed by quantifying inflammatory cytokine and chemokine expression using q-PCR and cell migration was assessed by a modified Boyden chamber migration assay. Our results demonstrate that eight ORs are expressed at basal levels in both airway and pulmonary macrophages, and that γ/LPS stimulation cooperatively increased this expression. Pulmonary macrophages exposed to the combined treatment of γ/LPS+octanal (an odorant) exhibited a 3-fold increase in MCP-1 protein production, compared to cells treated with γ/LPS alone. Supernatants from γ/LPS+octanal exposed macrophages also increased macrophage migration *in vitro*.

**Conclusions/Significance:**

Eight different ORs are expressed at basal levels in pulmonary macrophages and expression is upregulated by the synergistic action of γ/LPS. Octanal stimulation further increased MCP-1 production and the motility of macrophages. Our results suggest that ORs may mediate macrophage function by regulating MCP-1 production and cell migration.

## Introduction

Olfactory receptors (ORs) are one of the most ancient sensory systems in animals and are crucial for animal survival, procreation and evolution [1]–[4]. In mammals, ORs on the nasal olfactory epithelium detect scents by binding to odorant molecules [5], [6]. These ORs belong to the G-protein coupled receptor (GPCR) superfamily, the largest known superfamily of cell-surface receptors [7], [8]. Notably, ORs (like immune recognition receptors), are responsible for distinguishing molecules of immense diversity [1]. Although primarily associated with the nasal sensory systems, recent studies have demonstrated OR expression across a range of other tissues (e.g. such as testis, kidney, and heart) [9]–[12]. However, their function in non-olfactory tissues and cells remains largely unknown.

Interferon (IFN)-γ and lipopolysaccharide (LPS) play central roles in the activation of innate host defence pathways during infection [13], [14]. Previously, we have reported a unique signalling pathway that is activated cooperatively by IFN-γ and LPS. In a mouse model, we demonstrated that IFN-γ and LPS/Toll like receptor 4 (TLR4) pathways synergistically activate a novel steroid-resistant MyD88-dependent pathway in pulmonary macrophages [15], [16]. This leads to (i) production of the proinflammatory cytokine interleukin (IL)-27, (ii) glucocorticoid receptor (GR) dysfunction, and (iii) the development of airways hyperreactivity (AHR, enhanced airway smooth muscle contractility in response to spasmogenic stimuli) [15], [16]. This pathway may have significant implications for our understanding of how infection and immune molecules inhibit the endogenous glucocorticoid pathway to promote inflammation and smooth muscle constriction. Furthermore, we have demonstrated that this pathway may also contribute to the pathogenesis of infection-induced, steroid-resistant asthma [16]. In an attempt to further explore how IFN-γ and LPS regulate pulmonary macrophage function, we performed transcriptome profiling of the airways 12 hr after exposure to IFN-γ and LPS (γ/LPS). Surprisingly, exposure of the airways to γ/LPS resulted in markedly increased expression of a number of ORs. Subsequent investigations demonstrated that this OR expression was present on pulmonary macrophages. Therefore, we speculated that the increased OR expression induced by IFN-γ and LPS may play a functional role in innate immune responses of pulmonary macrophages, and as such, may contribute to the host defence process.

Interestingly, the concept that sensory receptors (both taste and smell receptors) contribute to host defence responses is beginning to emerge. Although examples are limited, both taste and smell receptors have been linked to the activation of resident cells in human and mouse airways that contribute to anti-microbial responses [17], [18]. For example, non-specific stimulation of ‘ectopically’ expressed taste receptors by flavonoids leads to increased ciliary beat frequency on airway epithelial cells and the relaxation of airway smooth muscle cells [17], [18]. Furthermore, activation of taste receptors in the human respiratory epithelium increases the production of nitric oxide (NO), resulting in enhanced mucociliary clearance and direct anti-microbial effects [19]. Intranasal application of female odorants in the lungs of male mice also stimulates leukocyte mobilization [20]. Collectively, these investigations suggest that the host defence system (including pulmonary macrophages) may employ sense receptors to respond to invading microbes.

As with other innate immune cells, pulmonary macrophages express a wide range of receptors, which enable them to recognize a wide variety of endogenous and exogenous stimuli [21]. These receptors collectively regulate differentiation, activation, migration, phagocytosis and cytotoxicity of macrophages [21], [22]. Thus, we speculated that ORs could also function to regulate one or more of these critical functions of macrophages in response to odorants. Macrophage migration to the site of infection is critical for microbial clearance and is regulated by a range of chemokines [23], [24]. Among these, monocyte chemotactic protein-1 (MCP-1) plays a central role [25], [26]. MCP-1 is primarily secreted by activated macrophages during the host defence response suggesting autocrine and paracrine functions [27]–[29].

In this study, we demonstrate OR expression in pulmonary macrophages and show that γ/LPS synergistically increase the expression of a number of ORs. Furthermore, we demonstrate that odorant-receptor stimulation greatly enhances both MCP-1 production and cell migration of γ/LPS-activated pulmonary macrophages. Thus, we propose that odorants produced by the metabolic activity of infecting microbes may activate ORs on macrophages leading to increased MCP-1 production and macrophage recruitment to the site of infection.

## Materials and Methods

### Mice

Wild type pathogen free BALB/c mice (6–8 wk) were obtained from the animal services unit of the University of Newcastle. All experiments were performed with approval from the animal ethics committee of the University of Newcastle (ethics approval number: A-2010-132).

### Administration of IFN-γ, LPS or γ/LPS

Mice were anesthetized (100 µl Alfaxan solution [1∶4] diluted with PBS i.v.) and the trachea was intubated with a 22-gauge catheter. Optimized doses of murine IFN-γ (1.5 µg/mouse; PeproTech, Rocky Hill, NJ), LPS (50 ng/mouse; Sigma-Aldrich, St Louis, MO) or γ/LPS (IFN-γ 1.5 µg+LPS 50 ng/mouse) in 50 µl vehicle (saline) were instilled intratracheally (i.t.) into the airways. Airway samples were then collected at 12 hr after treatment.

### Gene Chip Microarray

Airway tissues were collected by blunt dissection [15], [16]. The initial array experiment was performed on airway tissues (where macrophages may reside and are the primary tissues of the development of AHR) to get a global view of the transcriptional activity *in vivo* before moving to determine expression of molecules of interest in macrophages, where IFN-γ and LPS synergistically activate a novel steroid-resistant MyD88-dependent pathway. Airway tissues were disaggregated and homogenized and total RNA was extracted using the RNeasy Mini Kit (QIAGEN) as per the manufacturer’s instructions. For gene chip hybridization (Illumina microarray platform) and data analysis, RNA samples were stored in dry ice and shipped to the SRC Microarray Facility, The University of Queensland. The microarray data has been deposited into ArrayExpress (http://www.ebi.ac.uk/arrayexpress/). The accession number is E-MTAB-1893.

### Pulmonary Macrophage Isolation and Treatment

Pulmonary macrophages were isolated from mouse lungs according to previously described methods [29]. Briefly, mouse lung tissue was homogenized and single cell suspensions prepared. Macrophages were separated by density gradient centrifugation with Histopaque-1083 (Sigma-Aldrich, St Louis, MO) and plated at a concentration of 6×10^6^ cells/ml in Dulbecco’s Modified Eagle Medium (DMEM) containing 20% fetal calf serum (FCS). After 3 hr, 95% of adherent cells were macrophages, as confirmed by flow cytometry. Macrophages were cultured overnight, and then stimulated with different OR agonists, including amyl acetate (5 mM, Sigma-Aldrich, St Louis, MO), DL-α, ε-diaminopimelic acid (2.5 µM, MP Biomedicals, Santa Ana, CA), octanal (10 µM, Sigma-Aldrich, St Louis, MO) or vehicle (PBS or DMSO 0.1% v/v) for 12 hr. In some experiments, as indicated, macrophages were pre-treated with γ/LPS (IFN-γ 0.5 µg+LPS 50 ng/ml) for 12 hr before exposure to octanal.

### Preparation of Peritoneal Macrophages and Macrophage Migration Assay

Brewer’s thioglycolate (3 ml of 4% solution, BD Bacto, Franklin Lakes, NJ) was injected into the peritoneum of mice, and peritoneal-derived macrophages were isolated 4 days later, by washing the peritoneal cavity with 5 ml of ice-cold Hank’s buffered salt solution (HBSS). Erythrocytes were then eliminated using red cell lysis buffer as previously described and peritoneal-derived macrophage plated at 5×10^5^ cells/well in 6-well plates with complete Roswell Park Memorial Institute (RPMI) 1640 medium [15]. After 3 hr, non-adherent cells were washed away with PBS, and adherent cells were plated in fresh complete RPMI 1640 medium. Over 99% of adherent cells were macrophages, as determined by flow cytometry. For migration assays, 1×10^6^ peritoneal macrophages were seeded in the 24-well upper chamber of a cell culture insert with 8-µm pore-size membrane (BD Lifesciences, San Jose, CA). Macrophages were allowed to attached for 3 hr, then 600 µl of cell culture media with anti-MCP antibody (5 µ5f χε or isotype control antibody (Biolegend, San Diego, CA) or media containing MCP-1 (10 ng/ml) was added to the lower chamber and migrating macrophages were quantified in the lower chamber after 5 hr.

### Bone Marrow Derived Macrophage (BMDM) Culture and Polarization

Mouse femurs were flushed with 5 ml ice cold HBSS through a 70 µm cell strainer. After red blood cell lysis and washing with PBS, bone marrow cells were plated at 4×10^6^ cells per 10-cm dish with 10 ml of macrophage complete medium (L929 conditioned DMEM/12 medium). After culturing for 3 days, 5 ml of fresh medium was added to the culture. On day 7, floating cells were discarded and adherent cells used for further polarization studies. BMDM were polarized to M1 phenotype with IFN-γ (100 ng/ml) and LPS (10 ng/ml) or M2 phenotype with IL-4 (20 ng/ml) in the absence or presence of octanal. Polarized BMDM were then further stimulated with octanal (10 µM) or vehicle (DMSO, 0.1% v/v) for 12 hr.

### Quantitative Polymerase Chain Reaction (q-PCR)

Quantitative PCR was performed as described in detail elsewhere [15], [30]. Briefly, total RNA was isolated from treated pulmonary macrophages in TriReagent (Sigma-Aldrich, St Louis, MO) and reverse transcribed using Superscript III (Invitrogen, Carlsbad, CA). q-PCR was performed using an ABI Prism 7700 Sequence Detector (Applied Biosystems, Carlsbad, CA) using SYBR green reagents and expression was normalized to the house-keeping gene hypoxanthine-guanine phosphoribosyl transferase (HPRT). Primer sequences are shown in Table S1 and Table S2.

### Immunofluorescence Detection of OR622

Immunofluorescence assays were performed as described previously [16]. Briefly, macrophages isolated from mouse lungs were seeded on cover slips at 6×10^6^ cells/ml in DMEM containing 20% FCS. Cells attached to the slips were washed with cold PBS, fixed in 1% (w/v) paraformaldehyde in PBS buffer for 10 min at room temperature (RT), and blocked with 5% bovine serum albumin (BSA)/PBS for 1 hr at RT. Cells were then incubated with a polyclonal Ab to OR622 (antibodies-online, Atlanta, GA, USA) or isotype control (purified nonimmune rabbit IgG, Santa Cruz Biotechnology, Santa Cruz, CA) in 1% BSA/PBS overnight at 4°C. Cover slips were then washed with PBS and incubated with Cy3-conjugated goat anti-rabbit IgG (10 mg/ml; GE Healthcare, Buckinghamshire, U.K.) diluted in 1% BSA/PBS for 45 min in the dark at RT. Coverslips were then washed with PBS and stained with 4′,6-diamidino-2-phenylindole (DAPI) define (Sigma-Aldrich, St Louis, MO) for 10 min at RT. Slips were fixed on slides and visualized using a fluorescence microscope (BX51; Olympus, Tokyo, Japan) using a 100× objective lens. Fluorescence images were captured using a digital camera (DP70; Olympus, Tokyo, Japan). Mean fluorescence intensity was calculated and expressed as mean ± SEM.

### ELISA

Cell culture supernatants were collected after treatment as described and the MCP-1 ELISA kit (eBioscience, San Diego, CA) was used to detect MCP-1 protein levels according to manufacturer’s instructions.

### Macrophage Phagocytosis of Nontypeable *Haemophilus influenzae* (NTHi)

Isolation of pulmonary macrophages was performed as described above. Cells were then infected *in vitro* with 100 MOI NTHi for 8 hr as previously described [31]. To quantify intracellular bacteria load, macrophages were incubated with gentamicin (400 µg/mL) for 1 hr at 37°C to eliminate extracellular bacteria. After gentamicin treatment, cells were washed three times with PBS before being lysed with 0.25% saponin to release intracellular NTHi. To quantify extracellular bacteria numbers, supernatants were removed, plated onto chocolate agar plates and incubated overnight at 37°C in 5% CO_2_. Bacterial colonies were counted after incubation for 16 hr.

### Data Analysis

An initial one-way ANOVA was used to test for differences between groups. Values are presented as mean ± SEM for each experimental group. The number of mice ranged from 8 to 10 per group. Differences in means were considered significant if p was <0.05.

## Results

### Treatment with γ/LPS Up-regulates OR Expression Levels in Mouse Airway Tissue

**Figure 1:**
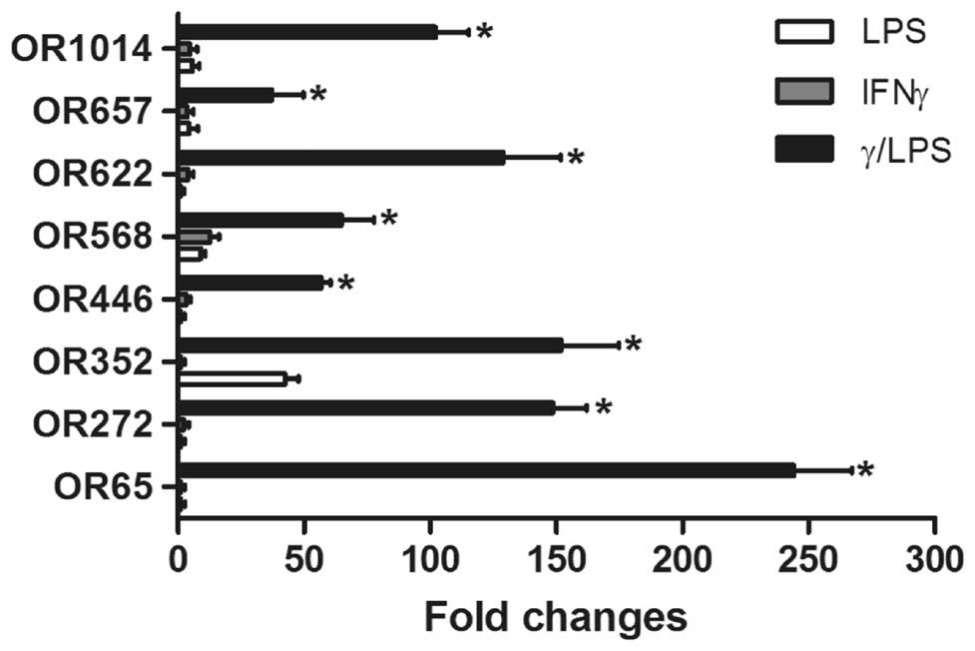
OR expression in the lung is induced by IFN-γ+LPS stimulation. Mice were intratracheally instilled with IFN-γ, LPS or γ/LPS, and airway samples were collected 12 hr later. Gene expression profiles were determined by microarray. Values are presented as mean ±SEM (n = 4), *P<0.05 (vs. other groups).

We have previously shown that pulmonary macrophages play a critical role in the development of γ/LPS-induced steroid-resistant inflammation and AHR by inhibiting endogenous glucocorticoid pathways [15], [30]. This suggests that these molecules activate pathways that enhance inflammation and host defence responses. Steroid-resistant inflammation and AHR develops 12 hr after exposure of the airways to these factors and is critically dependent on cooperative signalling [16]. To determine the pathways selectively activated by γ/LPS, we profiled mRNA expression in airway tissue by microarray 12 hr after exposure to LPS, IFN-γ and LPS+IFN-γ (γ/LPS). Interestingly, the data showed that a group of ORs (including of OR65, OR272, OR352, OR446, OR568, OR622, OR657 and OR1014) were significantly increased (Figure 1). Furthermore, the OR transcript levels following exposure to γ/LPS were profoundly elevated (approximately 50 to 250 fold increase) compared to groups exposed to IFN-γ or LPS alone. To further characterize OR expression, we quantified these receptors in compartments of the airways and in different tissues using q-PCR (Figure S1). The same OR receptors were expressed in the trachea, bronchi and parenchyma of the lung, albeit at levels lower than those in nasal mucosa, where ORs are typically studied. While expression levels were very low in the liver, a number of ORs were also highly expressed in the testis.

### γ/LPS Synergistically Enhance the Expression Levels of ORs in Pulmonary Macrophages

**Figure 2:**
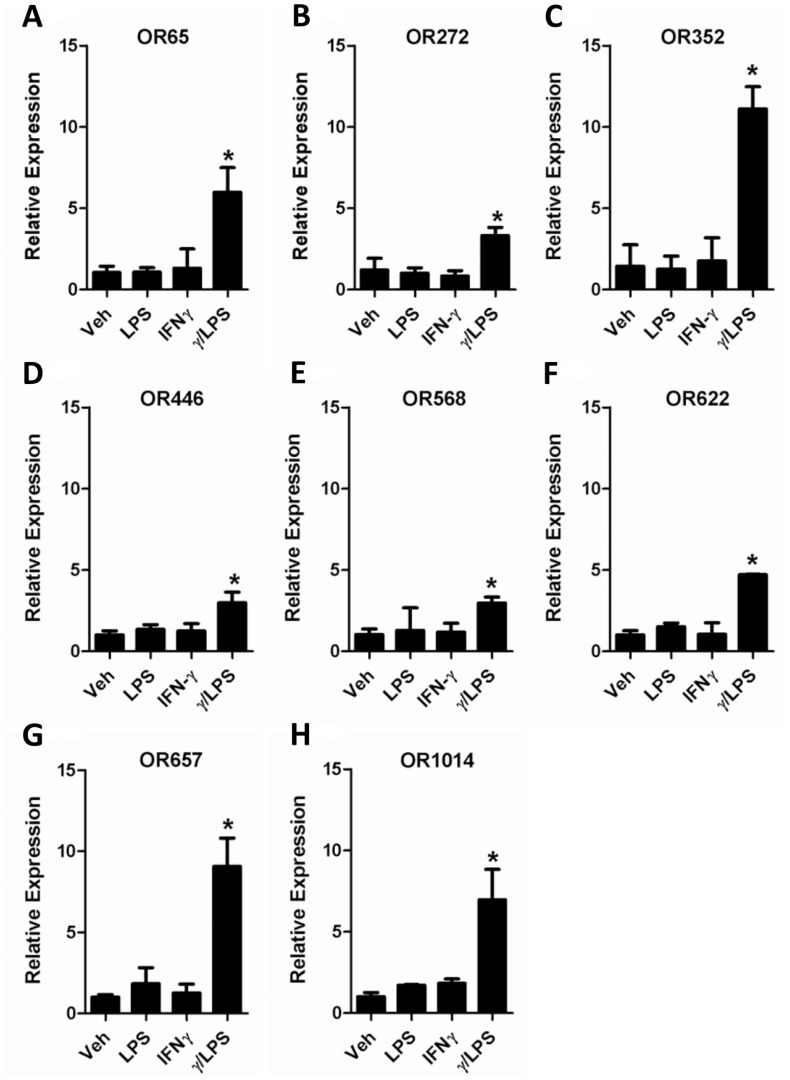
ORs are expressed by mouse pulmonary macrophages and upregulated by IFN-γ+LPS stimulation. Pulmonary macrophages were isolated by concentration gradient centrifugation and plated into 6-well plates. After 3 hr of attachment, adherent cells were treated with either IFN-γ, LPS or IFN-γ plus LPS (γ/LPS) for 12 hr. RNA was extracted and q-PCR was performed to assess OR gene expression. Values are presented as mean ±SEM (n = 3 separate experiments), *P<0.05 (vs. vehicle control).

**Figure 3:**
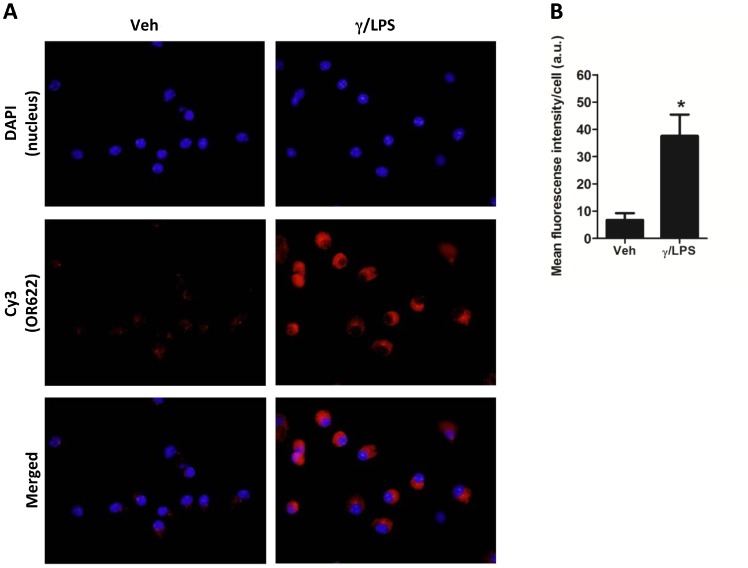
OR622 protein expression in mouse pulmonary macrophages. Pulmonary macrophages were isolated and attached to cover slides. After 3γ/LPS or vehicle control for 12 hr. OR622 protein expression was detected by immunofluorescence antibody staining. A) Representative cell images. Magnification×1000; blue = DAPI nuclear staining; red = Cy3-conjugated secondary antibody staining. B) Quantification of red fluorescence intensity (in arbitrary units, a.u), values are presented as means±SEM (n = 40 cells), *P<0.05 (vs. vehicle control).

Previously, we have shown that γ/LPS exposure activates pulmonary macrophages to induce steroid-resistant inflammation and AHR [15], [16]. We therefore examined whether OR expression occurred in pulmonary macrophages and whether levels could be synergistically and selectively upregulated by exposure to γ/LPS conditions *in vitro*. Pulmonary macrophages were isolated and treated with IFN-γ, LPS or γ/LPS and expression levels of the eight candidate ORs quantified by PCR. IFN-γ or LPS exposure alone failed to induce the expression of any of the eight ORs, compared with vehicle treatment (Figure 2). However, combined treatment with γ/LPS induced a significant upregulation of all eight OR transcripts (Figure 2). To further confirm OR expression in macrophages, we used the only commercially available antibody to any of the identified ORs (OR622) for immunofluorescence staining. Staining demonstrated that OR622 was expressed at low levels at baseline in pulmonary macrophages, and that combined treatment with γ/LPS dramatically increased OR622 antibody fluorescence intensity (Figure 3 A and B). Furthermore, confocal microscopy confirmed that OR622 is localized on the surface (Data not shown).

### Olfactory Agonists Stimulate MCP-1 Production by Pulmonary Macrophages

**Figure 4:**
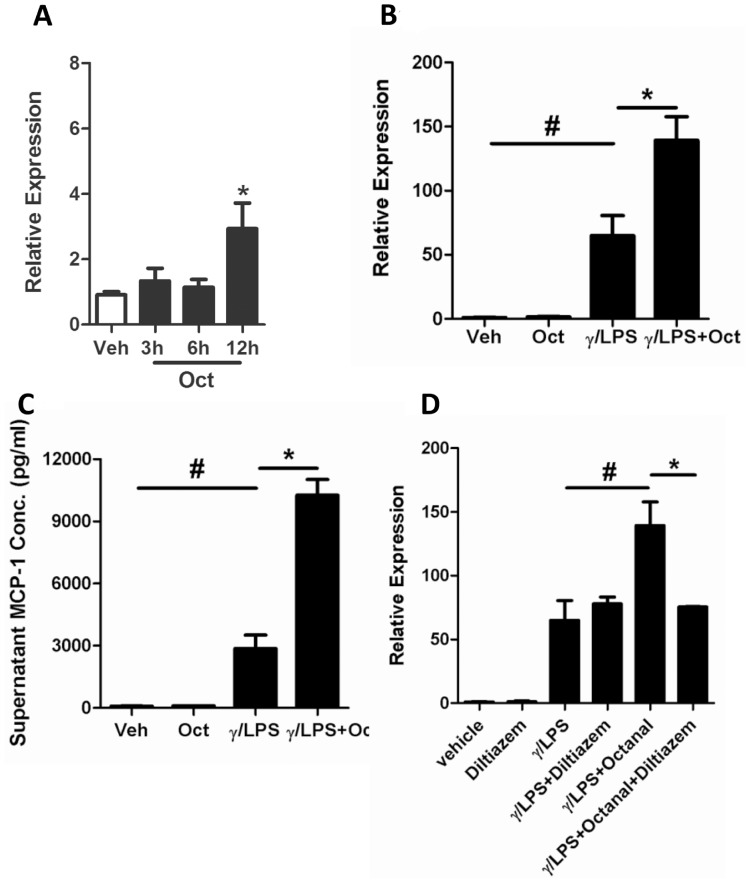
Octanal stimulation increases IFN-γ and LPS-induced MCP-1 expression in mouse pulmonary macrophages. Pulmonary macrophages were isolated and plated into 6-well plates. After 3 hr for attachment, adherent cells were stimulated with (A) octanal for 3 hr, 6 hr or 12 hr or (B/C) stimulated with octanal for 12 hr after exposure to γ/LPS (γ/LPS+Oct). MCP-1 mRNA expression was assessed by q-PCR and protein levels were determined by ELISA. In addition, (D) macrophages were stimulated with Octanal, γ/LPS or γ/LPS+Oct in the presence or absence of a diltiazem pre-treatment. Values are presented as mean ±SEM (n = 3), *P<0.05, #P<0.05.

Octanal is a model agonist used to stimulate OR activation and the first odorant where a structure-function relationship has been established [32]. To determine whether macrophage-expressed ORs are functional, we exposed pulmonary macrophages to octanal and assessed changes in macrophage function. Resting pulmonary macrophages were stimulated with 10 µM octanal for up to 12 hr and the expression levels of macrophage-derived chemokines (macrophage inflammatory protein 1(MIP-1), monocyte chemotactic protein-1 (MCP-1), monocyte chemotactic protein-2 (MCP-2), monocyte chemotactic protein-3 (MCP-3), chemokine (C-X-C motif) ligand 1(KC)), cytokines (tumor necrosis factor-α (TNF)-α, IFN-γ, interleukin-12 (IL-12)), and other proinflammatory factors (nitric oxide synthase-2 (NOS2), resistin-like molecule-α (FIZZ1), chitinase-3-like protein 3 (YM1) and arginase-1 (ARG1)) were quantified by q-PCR. Of the molecules assessed, MCP-1 mRNA expression was significantly increased following 12 hr of octanal exposure (Figure 4 A). There was no change in expression levels of other proinflammatory genes following octanal treatment (Figure S2). Next, we stimulated pulmonary macrophages with γ/LPS in combination with octanal for 12 hr to assess changes following γ/LPS-induced increases in OR expression. γ/LPS treatment alone resulted in approximately 65 and 32 fold increases in the levels of MCP-1 mRNA transcripts and protein in culture supernatants, respectively (Figure 4 B and C), as compared to vehicle exposure. The combined treatment with γ/LPS and octanal resulted in a further increase in MCP-1 levels, with approximately 140 and 114 fold increases in the levels of MCP-1 mRNA transcripts and protein in culture supernatants (Figure 4 B and C, respectively), as compared to vehicle.

As ORs are G-protein coupled receptors, we also investigated the role of Ca^2+^ flux in OR signalling using diltiazem (a Ca^2+^ ion channel blocker). Diltiazem has been previously used to inhibit OR function [33], [34]. Here, we showed that the addition of 50 µM diltiazem could eliminate the octanal- but not the γ/LPS-induced expression of MCP-1 (Figure 4 D), demonstrating a role for OR activation in increasing MCP-1 expression.

**Figure 5:**
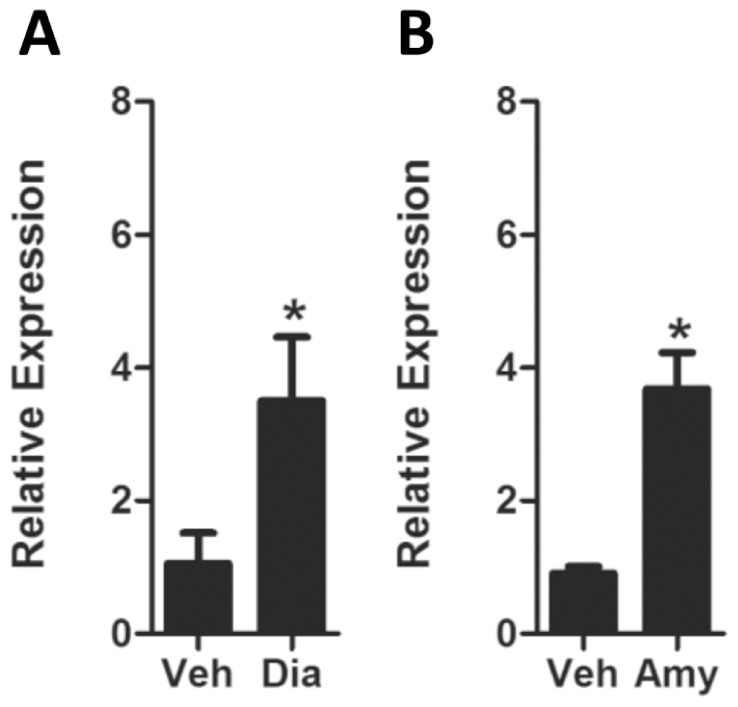
OR agonists induce MCP-1 expression in mouse pulmonary macrophages. Pulmonary macrophages were isolated and plated into 6-well plates. After 3 hr of attachment, adherent cells were stimulated with the OR agonists amyl acetate or diaminopimelic acid. Exposure to (A) amyl acetate or (B) diaminopimelic acid induced MCP-1 expression, as assessed by qPCR. Values are presented as mean ±SEM (n = 3 separate experiments), *P<0.05.

To further demonstrate that macrophage-expressed ORs are functional, we employed two other odorants -amyl acetate and diaminopimelic acid- to stimulate pulmonary macrophages. Amyl acetate is commonly used to activate ORs experimentally [35]. Diaminopimelic acid is a component of Gram-negative bacterial cell walls and an intermediate in the bacterial biosynthetic pathways for lysine and peptidoglycans, which has recently been identified to act as an OR agonist [36]. Exposure of resting pulmonary macrophages to each of these two OR agonists for 12 hr also resulted in increased MCP-1 expression (Figure 5).

### Octanal Exposure Promotes γ/LPS-induced MCP-1 Macrophage Migration

**Figure 6:**
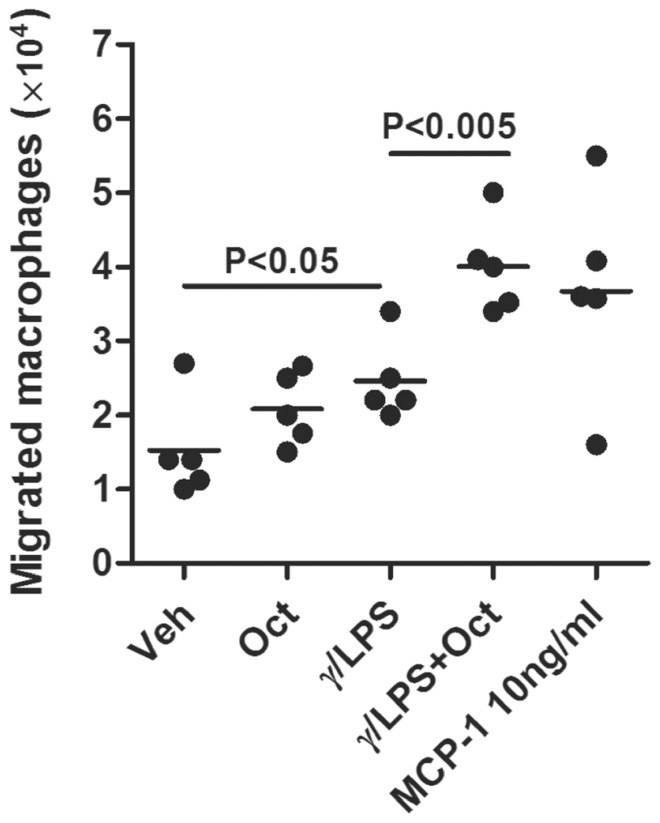
Supernatants from macrophage cultures stimulated with octanal, IFN-γ and LPS induce macrophage migration. Pulmonary macrophages were isolated and stimulated with octanal, γ/LPS or γ/LPS plus octanal (γ/LPS+Oct), and cell culture supernatants were collected. Additional peritoneal macrophages were prepared and plated into modified Boyden chambers. Stimulated culture supernatants were added to the lower chamber and macrophage migration was assessed (A). MCP-1- neutralizing antibody or control IgG were added into the stimulated culture supernatants and macrophage migration was assessed (B). Medium spiked with MCP-1 (10 ng/ml) was used as a positive control group. Values are presented as mean ±SEM (n = 3 separate experiments), P<0.05 was considered statistically significant.

To further characterize OR function on macrophages, we assessed whether γ/LPS-induced macrophage migration was enhanced by octanal-stimulated MCP-1 production using a chemotaxis chamber (Figure 6). Supernatants derived from macrophages treated with γ/LPS and octanal resulted in significantly enhanced macrophage migration (4.0±0.632×10^4^ cells/ml) compared to supernatants from γ/LPS treatment alone (2.5±0.555×10^4^ cells/ml) (Figure 6). We also compared migration rates to groups stimulated with recombinant MCP-1 (10 ng/ml) at the concentration of MCP-1 found in γ/LPS and octanal stimulated cultures (Fig. 4 C). Results indicated that supernatant from octanal and γ/LPS exposed cultures could induce macrophage migration at levels comparable to those induced by recombinant MCP-1 alone (10 ng/ml) (Figure 6 A). We further confirmed that the migration of macrophages was caused by the release of MCP-1 with a MCP-1-neutralizing antibody (Figure 6 B). Octanal-treated culture supernatants alone were unable to induce macrophage migration. These data demonstrate that the OR agonist octanal enhances macrophage migration, potentially by driving increased MCP-1 production in γ/LPS-treated pulmonary macrophages.

### Octanal does not Influence Macrophage Polarization

Next, we determined whether OR activation could effect the expression of markers that define the polarisation status of M1 or M2 macrophages or production of the anti-inflammatory cytokine IL-10. First, we assessed whether octanal altered the baseline expression of phenotypic markers of M1 and M2 cells in pulmonary macrophages. Exposure to octanal did not alter the expression NOS2 (M1 marker), or FIZZ1, YM1 or ARG1 (M2 markers) (Figure S3). γ/LPS exposure alone induced the expression of NOS2 in pulmonary macrophages, but not M2 cell markers, as previously described [29]. Octanal again had no effect on expression of these factors in the presence of γ/LPS exposure (Figure S3). We also generated BMDM (M0) and polarised them to the M1 or M2 phenotype (Figure S4). Again octanal had no effect on the expression of M1 or M2 markers. These data indicate that OR activation does not influence the polarization of macrophages.

### Octanal does not affect the Phagocytosis of NTHi by Pulmonary Macrophages

Finally, we assessed whether octanal exposure could alter the phagocytic capacity of pulmonary macrophages during bacterial infection. Macrophages were exposed to NTHi in the absence or presence of octanal and the rate of bacterial uptake was determined by a colony formation assay. Exposure to octanal did not alter the number of bacteria taken-up by macrophages (nor did it affect bacterial replication in culture medium). γ/LPS treatment of macrophages leads to rapid activation and exhaustion of these cells, which decreased their ability to clear bacteria from the supernatant. Octanal did not alter the effect of γ/LPS on bacterial clearance from the culture medium (Figure S5).

## Discussion

ORs are classically regarded as smell sense receptors that are predominantly expressed in the olfactory epithelium within the nasal cavity [1]. However, various studies have reported that ORs may also be expressed in non-olfactory tissues, where the roles of these ectopically expressed receptors remain unclear [9]. In the present study, we demonstrate that a group of ORs (OR65, OR272, OR352, OR446, OR568, OR622, OR657 and OR1014) are expressed in mouse airway tissue and pulmonary macrophages (Figure 1 and 2). We further demonstrate that IFN-γ and LPS stimulation act synergistically to enhance the expression of this group of ORs (Figure 2). We further demonstrate the presence of OR622 protein in pulmonary macrophages by immunofluorescence (Figure 3). Our data suggest that innate immune signals linked to the control of infection (IFN-γ and LPS) may regulate OR expression and suggest that these receptors may play a role in host defence responses, regulating macrophage function.

Although there are a limited number of studies on the role of ORs in immunity, a previous study demonstrated a role for odorants in activation of pulmonary immune response. Intranasal application of odorants derived from female mice stimulated IL-1β-independent activation of lung immunity and leukocyte mobilization in the lungs of male mice [37]. In our study, stimulation of ORs with the odorant agonists, octanal, amyl acetate and diaminopimelic acid, increased MCP-1 production in pulmonary macrophages (Figure 4 and 5). The effect appears to be selective for MCP-1, as no changes were observed in other major macrophage inflammatory mediators including NOS2, ARG1, FIZZ1, YM1, IL-10, MIP-1, MCP-2, MCP-3, KC, TNF-α, IFN-γ and IL-12 (Figure S2) [38]–[42]. Furthermore, supernatants from octanal-stimulated macrophages (with exposure to γ/LPS) significantly enhanced the migration of peritoneal-derived macrophages, at levels that were comparable to the levels observed by the addition of 10 ng/ml MCP-1 protein (Figure 6). As MCP-1 predominantly acts on macrophages, these data collectively suggest that the activation of ORs in pulmonary macrophages leads to increased production of MCP-1, which further amplifies macrophage migration during immune responses [43], [44].

Pulmonary macrophages are the most abundant innate immune cells in the lung and they are critical for maintaining pulmonary homeostasis, contributing to the clearance of foreign substances, elimination of infectious agents through phagocytosis and regulating aspects of innate and adaptive immune responses [45]–[47]. Broadly, macrophages can be classified into M1 and M2 subtypes based on their expression of specific markers [40], [47], [48]. M1 macrophages are associated with T helper type 1 (Th1) immune responses and contribute to the clearance of intracellular pathogens, killing of tumour cells and removal of tissue debris [40], [47], [49]. By contrast, M2 macrophages are associated with T helper type 2 (Th2) immune responses that underpin parasite eradication, suppression of inflammation and tissue remodelling [50]. We investigated whether octanal stimulation of ORs influences polarization of macrophages to specific phenotypes and phagocytosis of NTHi bacteria. Interestingly, octanal stimulation of bone marrow derived macrophages did not alter their M1/M2 polarisation phenotype. Furthermore, exposure of pulmonary macrophages to octanal did not alter the rate of phagocytosis or clearance of NTHi by pulmonary macrophages. These results imply that the roles of ORs expressed on macrophages are to facilitate the recruitment of these cells through the production of MCP-1, rather than directly altering their proinflammatory or innate host defence function.

Interestingly, many bacterial metabolites can act as OR agonists. For example, a recently identified odorant, diaminopimelic acid, is an intermediate product of the peptidoglycan and lysine biosynthetic pathways of bacteria [36]. This in conjunction with our data suggests that pulmonary macrophages have the potential to detect bacteria in their surroundings by sensing bacterial metabolites. Similarly, this notion is supported by the findings that *P. aeruginosa*-generated acyl-homoserine lactones (quorum-sensing molecules) activate taste receptors in human upper respiratory epithelium [51]. This pathogenic bacterium is commonly found in the lungs of patients with cystic fibrosis [51]. Although it is not clear at present if these quorum-sensing molecules can stimulate ORs, studies in *C. elegans* have shown that nematodes can respond to acyl-homoserine lactones through ORs expressed on their neuron cells [52], [53]. These studies suggest that bacterial quorum-sensing molecules not only directly stimulate taste receptors but also have the potential to activate the ORs of mammalian cells. Highly volatile compounds such as acetic acid, ethanol and 4-methylphenol are well-known odorant molecules that have also been identified in the culture of *P. aeruginosa* [54]. Interestingly, Fukuda and colleagues have recently shown that acetate (an acetic acid molecule lacking H^+^) released by *Bifidobacteria* protects the host from pathogenic infection through the activation of intestinal epithelial cells in a mouse model of *E. coli* O157 infection [55].

Our findings demonstrate that stimulation of macrophages with the bacterial bio-product LPS in conjunction with the host defence cytokine IFN-γ synergistically upregulates the expression of ORs. Activation of these ORs with the odorant octanal results in the increased expression and production of MCP-1 (but not other proinflammatory or host defence molecules). Although the fundamental roles of ORs on pulmonary macrophages needs to be further explored, we speculate that the ectopic expression of ORs on macrophages may serve as another pathogen-recognition pathway for the sensing of odorants linked to the metabolic activity of pathogenic bacteria. Thus, OR activation may act to promote the migration and accumulation of macrophages at the site of bacterial infection, where they can be further activated by specific pathogen recognition pathways (e.g. Toll like receptors).

## Supporting Information

Figure S1 **OR expression in different mouse tissues.** Tissue samples from naïve BALB/c mice were collected. RNA was extracted and gene expression was determined with q-PCR. Values are presented as mean ±SEM (n = 4 mice).
(TIF)

Figure S2 **Effects of macrophage OR activation on the expression of proinflammatory genes, chemokines and cytokines.** Pulmonary macrophages were isolated and plated into 6-well plates. After 3 hr of attachment, adherent cells were exposed to octanal for 12 hr. RNA was extracted and the mRNA expression of NOS2, ARG1, FIZZ1, YM1, IL-10, KC, MCP-2, MCP-3, MIP-1, TNF-α, IFN-γ and IL-12 was determined by q-PCR. Values are presented as mean±SEM (n = 3 separate experiments).
(TIF)

Figure S3 **OR activation has no effect on cultured pulmonary macrophage polarization.** Pulmonary macrophages were isolated and plated into 6-well plates. After 3 hr for attachment, adherent cells were exposed to either octanal alone or exposed to octanal after γ/LPS stimulation (γ/LPS+Oct). RNA was extracted and genes expression for NOS2, ARG1, FIZZ1 and YM1 were determined with q-PCR. Values are presented as mean ±SEM (n = 3).
(TIF)

Figure S4 **OR activation has no effect on cultured bone marrow derived macrophage polarization.** Bone marrow derived macrophages were prepared and polarized toward M1 or M2 type macrophages, before being stimulated with octanal for 12 hr. RNA was extracted and gene expression for the macrophage polarization marker genes NOS2, ARG1, FIZZ1 and YM1 were determined by q-PCR. Values are presented as mean ±SEM (n = 3).
(TIF)

Figure S5 **Exposure to OR agonist has no effect on macrophage phagocytic capacity for NTHi.** Pulmonary macrophages were prepared and exposed to NTHi in the absence or presence of octanal, and the rate of bacterial uptake was determined by colony formation assay. Colony formating units (CFU) of both intracellular (A) and extracellular (B) bacteria were counted. Values are presented as mean ±SEM (n = 3).
(TIF)

Table S1 **Primer sequences of ORs.**
(DOCX)

Table S2 **Primer sequences of other genes.**
(DOCX)
