# Supplementary figures and images for: Activation of Olfactory Receptors on Mouse Pulmonary Macrophages Promotes Monocyte Chemotactic Protein-1 Production
Source: PLoS One. 2013 Nov 21;8(11):e80148. doi: 10.1371/journal.pone.0080148 (PMC3836993; doi:10.1371/journal.pone.0080148)

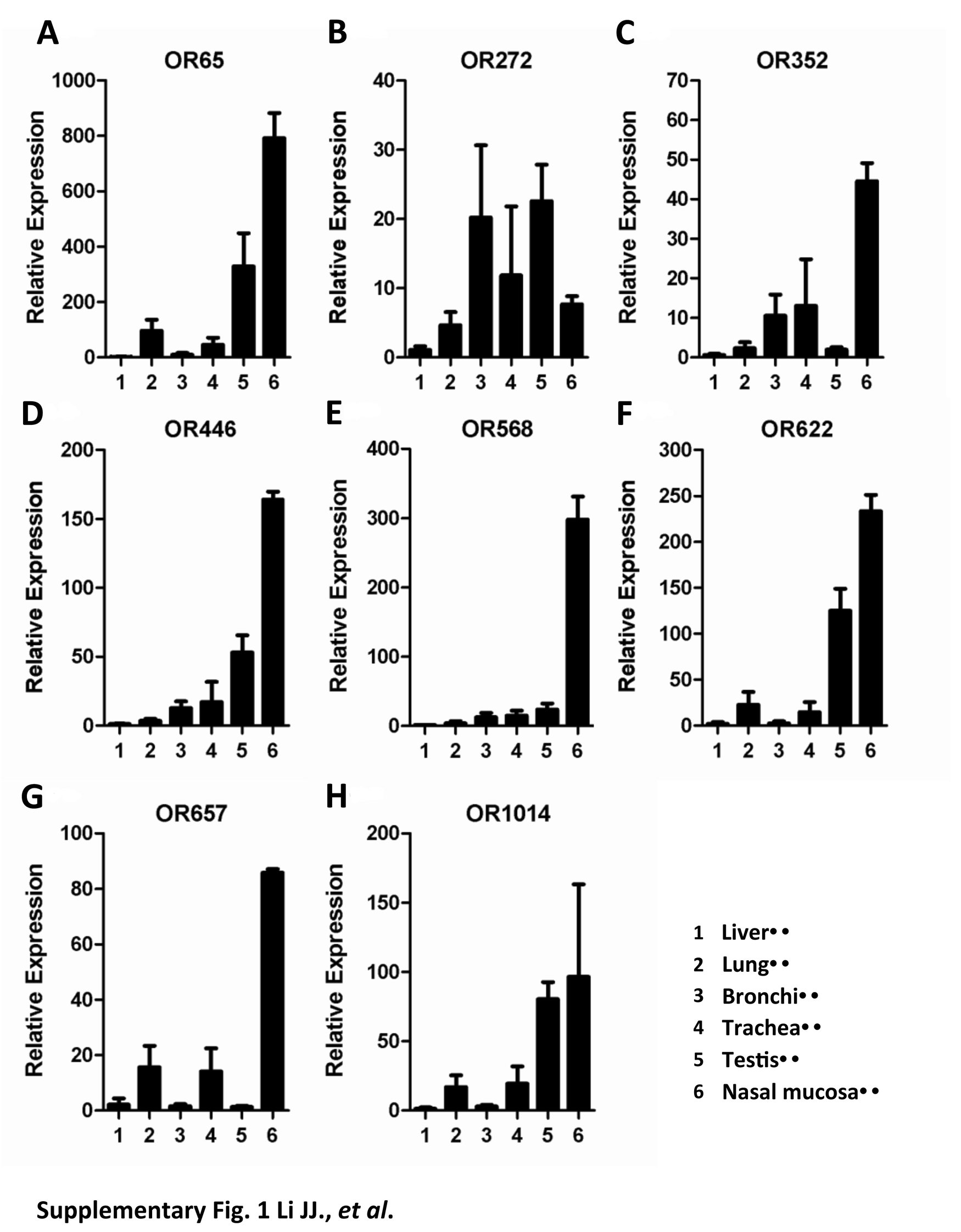

Supplement: Figure S1 — OR expression in different mouse tissues. Tissue samples from naïve BALB/c mice were collected. RNA was extracted and gene expression was determined with q-PCR. Values are presented as mean ±SEM (n = 4 mice). (TIF) [file pone.0080148.s001.tif]

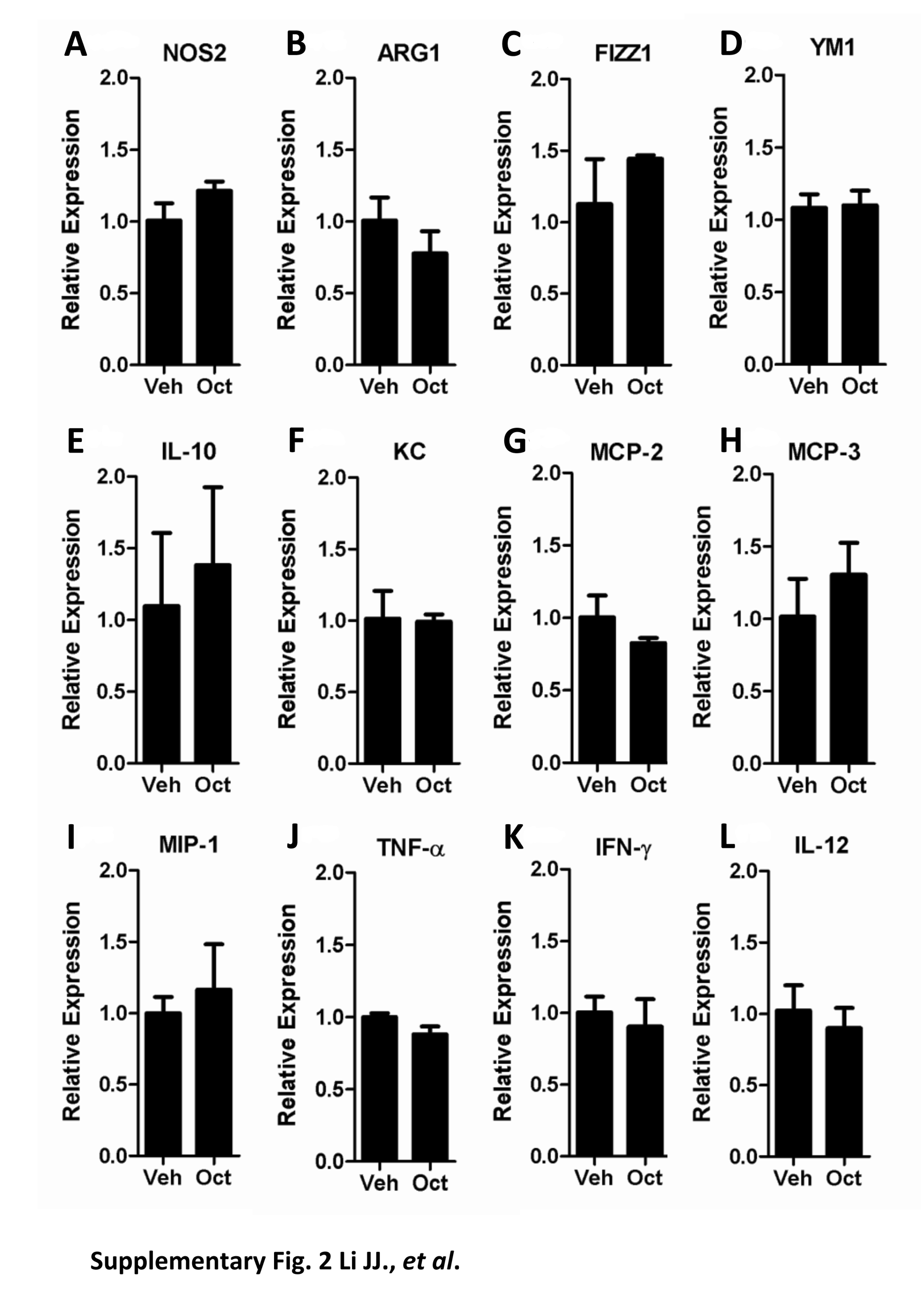

Supplement: Figure S2 — Effects of macrophage OR activation on the expression of proinflammatory genes, chemokines and cytokines. Pulmonary macrophages were isolated and plated into 6-well plates. After 3 hr of attachment, adherent cells were exposed to octanal for 12 hr. RNA was extracted and the mRNA expression of NOS2, ARG1, FIZZ1, YM1, IL-10, KC, MCP-2, MCP-3, MIP-1, TNF-α, IFN-γ and IL-12 was determined by q-PCR. Values are presented as mean±SEM (n = 3 separate experiments). (TIF) [file pone.0080148.s002.tif]

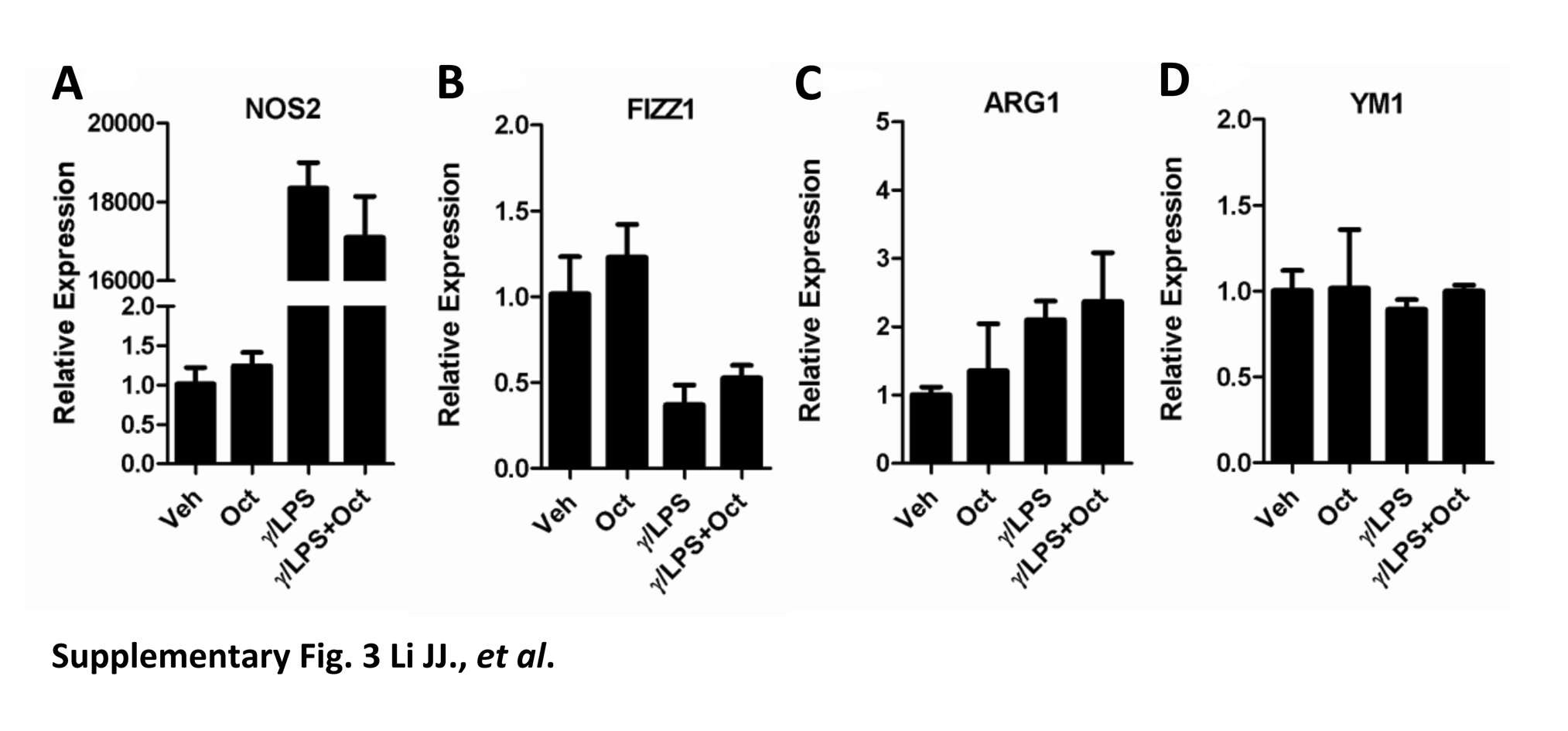

Supplement: Figure S3 — OR activation has no effect on cultured pulmonary macrophage polarization. Pulmonary macrophages were isolated and plated into 6-well plates. After 3 hr for attachment, adherent cells were exposed to either octanal alone or exposed to octanal after γ/LPS stimulation (γ/LPS+Oct). RNA was extracted and genes expression for NOS2, ARG1, FIZZ1 and YM1 were determined with q-PCR. Values are presented as mean ±SEM (n = 3). (TIF) [file pone.0080148.s003.tif]

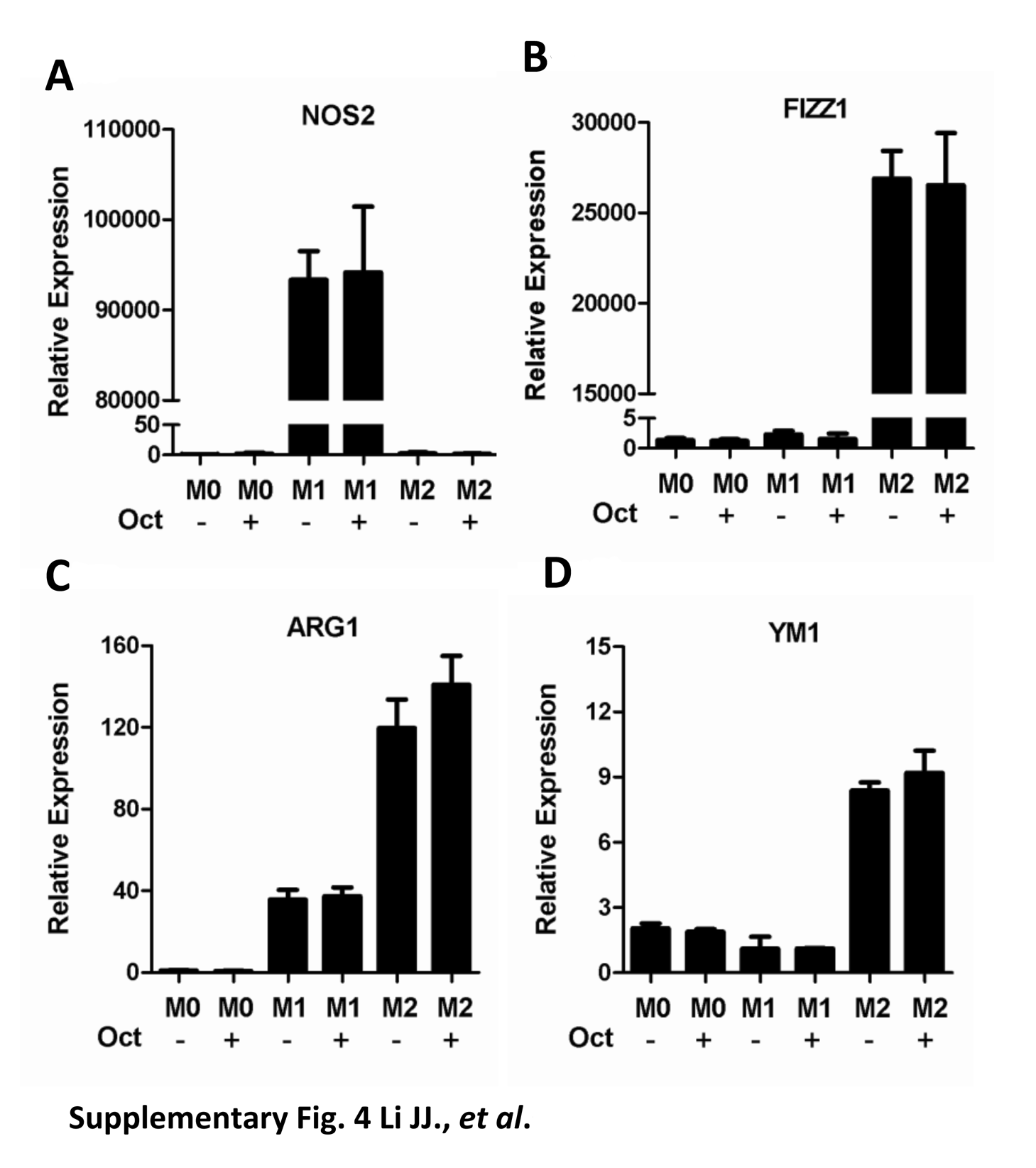

Supplement: Figure S4 — OR activation has no effect on cultured bone marrow derived macrophage polarization. Bone marrow derived macrophages were prepared and polarized toward M1 or M2 type macrophages, before being stimulated with octanal for 12 hr. RNA was extracted and gene expression for the macrophage polarization marker genes NOS2, ARG1, FIZZ1 and YM1 were determined by q-PCR. Values are presented as mean ±SEM (n = 3). (TIF) [file pone.0080148.s004.tif]

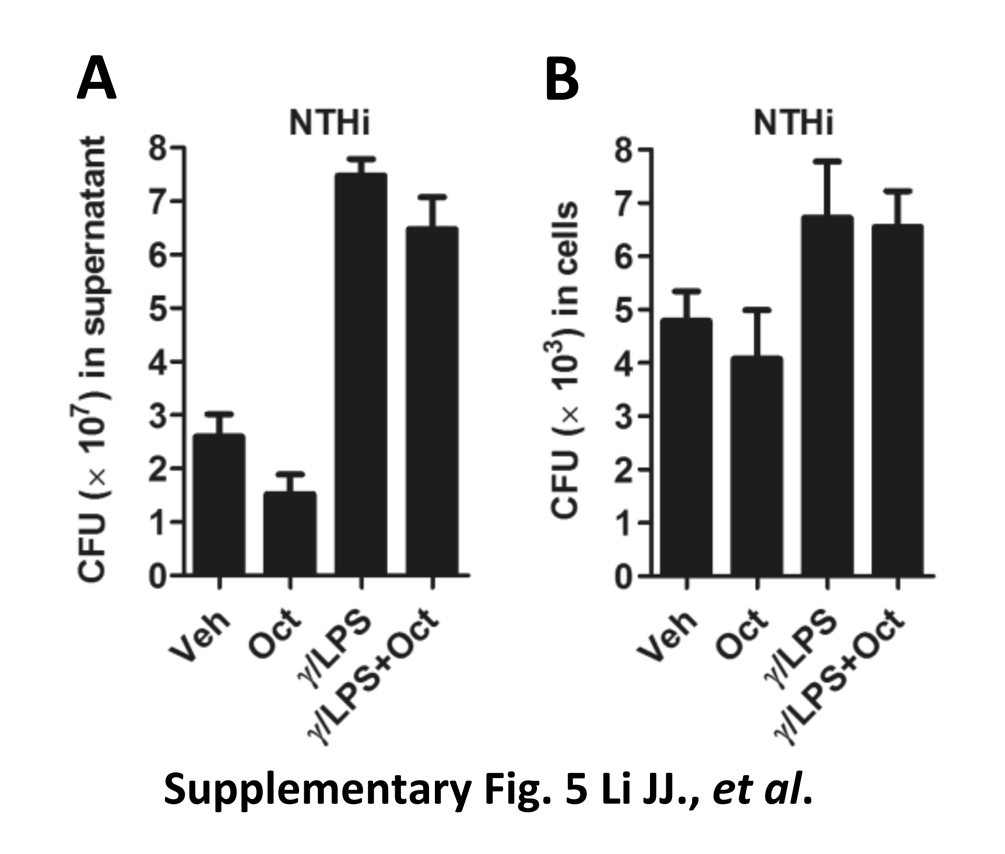

Supplement: Figure S5 — Exposure to OR agonist has no effect on macrophage phagocytic capacity for NTHi. Pulmonary macrophages were prepared and exposed to NTHi in the absence or presence of octanal, and the rate of bacterial uptake was determined by colony formation assay. Colony formating units (CFU) of both intracellular (A) and extracellular (B) bacteria were counted. Values are presented as mean ±SEM (n = 3). (TIF) [file pone.0080148.s005.tif]
