# Supplementary material for: Activation of Olfactory Receptors on Mouse Pulmonary Macrophages Promotes Monocyte Chemotactic Protein-1 Production
Source: PLoS One. 2013 Nov 21;8(11):e80148. doi: 10.1371/journal.pone.0080148 (PMC3836993; doi:10.1371/journal.pone.0080148)
Supplement: Table S1 — Primer sequences of ORs. (DOCX) [file pone.0080148.s006.docx]

**Table S1.** Primer sequences of ORs.

| OR65 | Forward | 5′ AGGACCGCTGATCATCCACACAGCA3′ |
| --- | --- | --- |
|  | Reverse | 5′ AAGGAGGGTGCCGTTGCCAAGT3′ |
| OR272 | Forward | 5′ ACCTGCTCTGCCCACCTGACAGT3′ |
|  | Reverse | 5′ TGAGGGCCTCCATCACGCTTTGA3′ |
| OR352 | Forward | 5′ TTGCCCATCTCTCTTTCAGTGGGGG3′ |
|  | Reverse | 5′ TGAGAGCCACACGTGGACAAGGC3′ |
| OR446 | Forward | 5′ CTGGAGATGCTCCTGTTTGGGCTGT3′ |
|  | Reverse | 5′ TCCAGGCAGATGAGCCCCAGGATAA3′ |
| OR568 | Forward | 5′ GGCCTGAGAGCAGCTCAGGTCTG3′ |
|  | Reverse | 5′ TGGCCAGCAGAACTCCGGATTCC3′ |
| OR622 | Forward | 5′ TCTGCAGCTCTGAAGATGCTTGGCA3′ |
|  | Reverse | 5′ GGGCATGGCAAAACCATGAGCACA3′ |
| OR657 | Forward | 5′ GCTGGCAGCATTGGCTCTCCCTACC3′ |
|  | Reverse | 5′ GGCAAGGCCCATGTCCACCACAGAG3′ |
| OR1014 | Forward | 5′ ACCCTGTCATGCAGCTGGTTCTGC3′ |
|  | Reverse | 5′ GCCACACAGCCAGAAAAGGAG ATGC3′ |
