# Supplementary material for: Activation of Olfactory Receptors on Mouse Pulmonary Macrophages Promotes Monocyte Chemotactic Protein-1 Production
Source: PLoS One. 2013 Nov 21;8(11):e80148. doi: 10.1371/journal.pone.0080148 (PMC3836993; doi:10.1371/journal.pone.0080148)
Supplement: Table S2 — Primer sequences of other genes. (DOCX) [file pone.0080148.s007.docx]

**Table S2.** Primer sequences of other genes.

| HPRT | Forward | 5′ AGGCCAGACTTTGTTGGATTTGAA 3′ |
| --- | --- | --- |
|  | Reverse | 5′ CAACTTGCGCTCATCTTAGGCTTT 3′ |
| IL-10 | Forward | 5′ CATTTGAATTCCCTGGGTGAGAAG3′ |
|  | Reverse | 5′ GCCTTGTAGACACCTTGGTCTTGG3′ |
| IFN-γ | Forward | 5′ TCTTGAAAGACAATCAGGCCATCA3′ |
|  | Reverse | 5′ GAATCAGCAGCGACTCCTTTTCC3′ |
| TNF-α | Forward | 5′ GTCTACTGAACTTCGGGGTGATCG3′ |
|  | Reverse | 5′ AGCCTTGTCCCTTGAAGAGAACCT3′ |
| IL-12 | Forward | 5′ CTGTGCCTTGGTAGCATCTATG3′ |
|  | Reverse | 5′ GCAGAGTCTCGCCATTATGATT3′ |
| NOS2 | Forward | 5′ CCTCGGAGGTTCACCTCACTGT3′ |
|  | Reverse | 5′ CAGAAACTTCGGAAGGGAGCAAT3′ |
| ARG1 | Forward | 5′ GCTCCAAGCCAAAGTCCTTAGAGAT3′ |
|  | Reverse | 5′ AGGAGCTGTCATTAGGGACATCAAC3′ |
| MR | Forward | 5′ GCCATGAGGCTTCTCCTGCTTCTG3′ |
|  | Reverse | 5′ TGCCGTCTGAACTGAGATGGCACT3′ |
| FIZZ1 | Forward | 5′ TACTTGCAACTGCCTGTGCTTACT3′ |
|  | Reverse | 5′ TATCAAAGCTGGGTTCTCCACCTC3′ |
| YM1 | Forward | 5′ GACAGGCCAATAGAAGGGAGTTTCA3′ |
|  | Reverse | 5′ GACGGTTCTGAGGAGTAGAGACCAT3′ |
| KC | Forward | 5′ CAATGAGCTGCGCTGTCAGTG3′ |
|  | Reverse | 5′ CTTGGGGACACCTTTTAGCATC3′ |
| MCP-1 | Forward | 5′ AGAGCCAGACGGGAGGAAG3′ |
|  | Reverse | 5′ CCAGCCTACTCATTGGGATC3′ |
| MCP-2 | Forward | 5′ GGGTGCTGAAAAGCTACGAG3′ |
|  | Reverse | 5′ TTCCAGCTTTGGCTGTCTCT3′ |
| MCP-3 | Forward | 5′ AATGCATCCACATGCTGCTA3′ |
|  | Reverse | 5′ CTTTTGGAGTTGGGGTTTTCA3′ |
| MIP-1 | Forward | 5′ CCTCTGTCACCTGCTCAACA3′ |
|  | Reverse | 5′ GATGAATTGGCGTGGAATC3′ |
